# Relationship between helicobacter pylori infection and pityriasis versicolor:can helicobacter pylori infection be a new etiologic factor for pityriasis versicolor?

**DOI:** 10.3906/sag-1910-48

**Published:** 2020-06-23

**Authors:** Ömer KUTLU, Zeynal DOĞAN, Hatice Meral EKŞİOĞLU, Murat KEKİLLİ

**Affiliations:** 1 Department of Dermatology and Venereology, School of Medicine, Uşak University, Uşak Turkey; 2 Department of Gastroenterology, Adıyaman Training and Research Hospital, Adıyaman Turkey; 3 Department of Dermatology and Venereology, Ankara Training and Research Hospital, Ankara Turkey; 4 Department of Gastroenterology, Ankara Training and Research Hospital, Ankara Turkey

**Keywords:** Helicobacter pylori, Malassezia, pityriasis versicolor, tinea versicolor, urea breath test

## Abstract

**Background/aim:**

H. pylori has been found to be related to certain dermatological diseases. However, there is no data as yet to propose an association between H. pylori and pityriasis versicolor. In this study, we aimed to evaluate the association between H. pylori and pityriasis versicolor.

**Materials and methods:**

This was a prospective study performed in the Gastroenterology and Dermatology and Venereology departments of the Health Sciences University, Ankara Training and Research Centre. A total of 57 consecutive patients (27 pityriasis versicolor, 30 telogen effluvium) were enrolled from the Department of Dermatology and Venereology. All patients were screened for H. pylori IgG and CagA. In addition, urea breath test was carried out to detect the existence of H. pylori infection.

**Results:**

There were significantly higher rates of H. pylori positivity, H. pylori IgG in serum in the pityriasis versicolor group compared to the telogen effluvium group (P < 0.05). In addition, the number of patients with dyspeptic complaints was higher in the pityriasis versicolor group than in the telogen effluvium group. The odds ratio for dyspepsia, H. pylori positivity, and H. pylori IgG were 2.48, 1.67, and 1.78, respectively.

**Conclusion:**

In this study, we found a statistically significant relationship between H. pylori infection and pityriasis versicolor. Therefore, H. pylori eradication could be considered in recurrent pityriasis versicolor patients with dyspepsia. New studies are required to clarify the effect of eradication treatment on the clinical course of pityriasis versicolor.

## 1. Introduction

Helicobacter pylori is a bacterium in the gastric and duodenal mucosa that causes dyspeptic symptoms and gastric cancer [1]. H. pylori has been found to be related to certain dermatological diseases such as chronic spontaneous urticaria, atopic dermatitis, lichen planus, and vitiligo [2,3].

Pityriasis versicolor (PV) also known as tinea versicolor is a chronic, benign, and asymptomatic infection caused by Malassezia spp. [4]. Hereditary factors, hyperhidrosis, seborrhoea, and the chemical constitution of sebum may play an important role in the aetiology of PV [5].

 H. pylori-induced systemic inflammation has been shown to play a role in the pathogenesis of skin diseases such as chronic urticaria, and atopic dermatitis leading to dysregulation of cytotoxic and other cell-mediated mechanisms in the skin microenvironment [6]. This immune dysregulation in the skin microenvironment could provide the basis for the formation of pityriasis versicolor (PV) in patients with H. pylori infection. The aim of this research was to investigate the relationship of H. pylori with skin diseases in patients with PV.

## 2. Materials and methods

### 2.1. Study population

This was a prospective study performed in the Gastroenterology and Dermatology and Venereology Departments of the Health Sciences University, Ankara Training and Research Hospital. Twenty-seven consecutive patients with PV were enrolled from the Department of Dermatology and Venereology. The diagnosis of these patients was done by clinical examination, Wood’s lamp, and KOH examination. The control group consisted of 30 consecutive patients who presented to the Department of Dermatology and Venereology due to telogen effluvium (TE). The TE group was described as patients who had hair loss occurring 3 months after a triggering event and increasing telogen hair over 20% in a trichogram. Since there is no immunological basis for TE, the control group was selected as those with TE. The diagnosis of patients with PV and TE was performed by the department of Dermatology and Venereology. 

The presence of H. pylori was determined by H. pylori IgG, H. pylori CagA, and urea breath test (UBT).

### 2.2. Antibody measurements

 H. pylori IgG and H. pylori CagA were detected by Elisa (Dia. PRO. Diagnostic Bioprobes Srl. Milano, Italy). Samples with a concentration higher than 20 U/mL were considered positive for H. pylori and 5 arbU/mL for anti CagA.

### 2.3. C14 urea breath test

Urea breath test was conducted as follows: after a night-time fast, the patients received a 14C-labelled urea-containing drug (HelicapTM, Institute of Isotopes, Budapest, Hungary) with 50 mL water. After 15 min, the patients exhaled into a dry cartridge (Heliprobe breath card, Kibion AB, Uppsala, Sweden) until the colour of the card indicator changed from orange to yellow, which took about 1–2 min. Finally, the test results were displayed on at screen analyser that indicates radioactivity as counts per minute (CPM). 50 CPM: patient infected. 25 CPM: patient not infected; 25–50 CPM, borderline or suspicious outcome. We considered <25 CPM as a negative result and considered >50 CPM as a positive result.

Patients receiving proton pump inhibitor therapy for the last 1 month, who had a history of H. pylori eradication, who were smoking, drinking alcohol, who were pregnant, who had a major psychiatric disorder, candidiasis and being over-eager were excluded from this study. Patients were interrogated for the presence of dyspeptic symptoms. Dyspepsia was described as swelling, upper gastrointestinal discomfort, regurgitation, premature sense of fullness and heartburn. Ethical approval for this study was given by the hospital ethics committees with 0558-13.08.14 and informed written consent was acquired from all patients.

### 2.4. Statistical analysis

Chi-square test was used for categorical data differences between the 2 groups. A P-value of <0.05 was considered statistically significant. The odds ratio (OR) was obtained to quantify the strength of the association between case and control groups. All statistical analyses were performed using SPSS 20 statistical package (IBM Corp., Armonk, NY, USA).

## 3. Results

Ten patients were excluded from this study since they had suspicious results in UBT. Fifty-seven patients (41 female, 16 male patients) participated in the study. Twenty-seven patients had PV while 30 patients had TE. 

When the demographic characteristics of the patients were evaluated, there was no significant differentiation between the ages and body mass indexes of PV and control groups. The Hb score in the TE group was statistically lower than in the PV group (P < 0.05). There were no differences between the 2 groups regarding socioeconomic status and smoking habit. Demographic and laboratory characteristics of the patients are shown in Table 1.

**Table 1:** Demographic and laboratory characteristics of pityriasis versicolor and telogen effluvium groups.

Characteristic	Pityriasis versicolor(n = 27)	TE group(n = 30)	P value
Age (years)	29.52 ± 12.76	29.20 ± 12.79	0.816
Height (m)	166.59 ± 8.92	159.11 ± 5.30	0.031
Body mass (kg)	70.07 ± 13.71	61.94 ± 11.51	0.816
BMI (m/kg2)	25.18 ± 4.11	24.57 ± 5.09	0.648
Hb (g/dL)	14.26 ± 1.63	13.32 ± 1.26	0.022
WBC (×109/L)	7.49 ± 1.60	7.02 ± 1.58	0.291
PLT (×109/L)	275.82 ± 79.56	248.30 ± 72.18	0.221
ALT (IU/L)	20.93 ± 14.95	18.11 ± 10.15	0.798
AST (IU/L)	22.07 ± 9.83	21.11 ± 4.54	0.660
Urea mg/dL	28.41 ± 6.79	24.89 ± 6.09	0.175
Creatinine mg/dL	0.91 ± 0.17	0.85 ± 0.08	0.421

n: number of patients TE: Telogen effluvium

Dyspeptic complaints, H. pylori IgG, and UBT positivity were found to be significantly higher in the PV group than in the TE group (P < 0.05). However, no significant differentiation was found between the 2 groups in terms of H. pylori CagA antigen levels (P > 0.05). (Table 2). Odds ratio for dyspepsia, UBT, and H.pylori Ig G positivity were 2.48, 1.67, and 1.78, respectively (Table 3).

**Table 2:** Laboratory results of H. pylori and dyspepsia positivity in patients with pityriasis versicolor and telogen effluvium.

	PV* group	TE group	χ2 p*	OR
Dyspepsia positivity	19 (70.4%)	8 (26.7%)	0.001	2.48(1.3–4.6)
H. pylori positivity	18(66.6%)	10(33.3%)	0.040	1.67 (1.0–2.28)
CagA positivity	17(63.0 %)	17(56.7%)	0.788	1.13(0.69–1.85)
H. pylori IgG positivity	24(88.9%)	19(63.3%)	0.033	1.78(1.15–2.74)

*P ≤ 0.05 = Statistically significant. Chi‑square test was performed. OR: Odds ratio

**Table 3:** Odds ratio for dyspepsia, UBT, and H. pylori IgG positivity.

Characteristic	OR
Dyspepsia positivity	2.48 (1.3–4.6)
H. pylori positivity	1.67 (1.0–2.28)
H. pylori IgG positivity	1.78(1.15–2.74)

OR: Odds ratio (minimum, maximum)

## 4. Discussion

An increasing number of dermatologic diseases including atopic dermatitis, aphthous stomatitis, rosacea, Henoch-Schoenlein purpura, alopecia areata, vitiligo, and psoriasis have been associated with H. pylori infection since the identification of H. pylori in 1983. In addition to its direct injury to target tissues, H. pylori could use its destructive effects indirectly by interfering with the immune system in these diseases [7]. 

Chronic urticaria and immune thrombocytopenic purpura are the skin diseases most associated with H. pylori infection [8,9]. Of note, some studies suggested that eradication of H. pylori is related to improvement of skin diseases [10–12]. In this context, illumination of the association between H. pylori infection and skin diseases may contribute to the prevention or treatment of these diseases. Although there are many studies on the association between H. pylori infection and skin disease, according to our knowledge, there is no data on H. pylori infection and PV. 

Superficial fungal infections of the skin are common diseases in all parts of the world [13]. Pityriasis versicolor is a noninflammatory superficial fungal infection of the skin caused by fungi of the Malassezia type, that can lead to social, emotional, and economic problems for patients [14]. The diagnosis of PV is confirmed by the use of Wood’s lamp, KOH examination under the microscope, and fungal cultures, in addition to presence of characteristic clinical changes [15–17]. Risk factors that can predispose an individual to PV include humid environment, malnutrition, and immunosuppression such as with HIV or renal transplant disease, corticosteroid use [18,19]. 

Helicobacter pylori infection has been found to be associated with mucosal inflammation due to infiltration by monocytes and neutrophils in the gastric mucosa. Furthermore, translocation of CagA into gastric epithelial cells causes elevated levels of proinflammatory cytokines including tumour necrosis factor–α, interleukin (IL)-6, IL-8, IL-10, and IL-17 [20,21]. Immune dysregulation due to H. pylori infection can change the skin microenvironment and might contribute to the tendency to PV. 

In this study, we found that H. pylori IgG and UBT positivity were significantly higher in patients with PV than the control group, 1.78 and 1.67 times, respectively. Furthermore, dyspepsia was present much more in patients with PV compared to the control group. The odds ratio for patients with PV for dyspepsia was 2.48. In this regard it can be speculated that acute and chronic inflammation due to previous H. pylori infection may lead to a tendency to PV, and H. pylori could be a new etiologic factor for PV.

CagA strains of H. pylori are prone to more severe gastrointestinal diseases as well as autoimmune systemic diseases including idiopathic thrombocytopenic purpura, autoimmune thyroiditis as well as vitiligo [2,22,23]. The CagA protein stimulates the gastric epithelium to secrete high levels of inflammatory cytokines such as IL-8, macrophage inflammatory protein (MIP-1α), and IL1-β. These cytokines have chemotactic activity on neutrophils, T lymphocytes, and other leukocytes [20]. We did not find any relationship between H. pylori infection and PV in terms of CagA positivity. In this regard, it can be speculated that high CagA levels trigger autoimmunity rather than immunosuppression. Therefore, it can be concluded that CagA levels could be normal in patients with PV. 

The UBT test, which has a specificity of 93%–100% and a sensitivity of 88%–96%, allows the determination of H. pylori status more precisely [24]. In this study, we carried out the UBT test to provide better evidence supporting whether active infection with H. pylori may trigger PV or not. We detected that UBT positivity was higher in patients with PV. H. pylori infection, which secretes the VacA protein, affects macrophages and B- and T-lymphocytes and leads to decreased IL-2 production with the consequence of suppressed IL-2-mediated T lymphocyte proliferation [20,21]. This finding may suggest that active H. pylori infection, which can be shown by UBT, may decrease proinflammatory cytokines and may lead to immunosuppression. 

In our study, the prevalence of H. pylori detected by UBT was lower than the general population which can be explained in several ways. Eighteen out of 27 patients (66.6%) with PV and 10 out of 33 (33.3%) patients with TE had H. pylori positivity. If patients with PV and TE were considered totally, H. pylori was positive in 28 out of 57 (49%) patients presented to the dermatology clinic due to PV and TE without dyspepsia as the main complaining. In addition, the prevalence of H. pylori differs in terms of the development of socioeconomic and infrastructure in countries, cities as well as various regions of the same city. The population participating in our study was one of the highest socioeconomic cities in Turkey. Furthermore, it is well established that as the levels of development increases in a country, the prevalence of H. pylori decreases. Özden et al. reported the prevalence of H. pylori as 78.5% in 1990 and 66.3% in 2000 [25]. This result is clearly related to the improvement of the socio-economic level and infrastructure. Therefore, we believe that the prevalence of H. pylori has decreased significantly over time due to improvements in socioeconomic status and infrastructure during the last 15 years. Lastly, according to our study, despite the fact that the ratio of the H. pylori positivity was close to the prevalence of the general population, this prevalence would be slightly higher if the number of patients had higher.

Although previous studies mostly detected an association between autoimmune skin disease and H. pylori infection, there are few studies on H. pylori infection and infectious skin diseases. In this study, we found a statistically significant relationship between H. pylori infection and PV. Therefore, H. pylori eradication could be considered in PV patients concomitant with dyspepsia. The relationship between H. pylori infection and PV should be supported by further studies with a larger sample size. In addition, new studies are required to clarify the effect of eradication treatment on the clinical course of PV. 

## Acknowledgements

The authors received no financial support for the research and/or authorship of this article.
